# Short-Term Exposure to Wood Smoke Increases the Expression of Pro-Inflammatory Cytokines, Gelatinases, and TIMPs in Guinea Pigs

**DOI:** 10.3390/toxics9090227

**Published:** 2021-09-20

**Authors:** Carlos Ramos, Rebeca Cañedo-Mondragón, Carina Becerril, Georgina González-Ávila, Ana Laura Esquivel, Ana Lilia Torres-Machorro, Martha Montaño

**Affiliations:** 1 Laboratorio de Biología Celular, Departamento de Investigación en Fibrosis Pulmonar, Instituto Nacional de Enfermedades Respiratorias Ismael Cosío Villegas (INER), Calzada de Tlalpan 4502, Colonia Belisario Domínguez Sección XVI, Alcaldía Tlalpan, Mexico City 14080, Mexico; carlos.ramos26@yahoo.com.mx (C.R.); anarebeca_cm@hotmail.com (R.C.-M.); lcbb6@hotmail.com (C.B.); ana.torres@iner.gob.mx (A.L.T.-M.); 2 Laboratorio de Oncología Biomédica, Instituto Nacional de Enfermedades Respiratorias Ismael Cosío Villegas (INER), Calzada de Tlalpan 4502, Colonia Belisario Domínguez Sección XVI, Alcaldía Tlalpan, Mexico City 14080, Mexico; ggonzalezavila@yahoo.com; 3 Departmento de Sistemas Biológicos, Universidad Autónoma Metropolitana—Unidad Xochimilco (UAM-X), Mexico City 04960, Mexico; valuzza@hotmail.com

**Keywords:** wood smoke, cytokines, inflammation, extracellular matrix remodeling, matrix metalloproteinase, tissue inhibitor of metalloproteinase

## Abstract

Exposure to air pollutants in wildfire smoke and indoor pollution causes lung diseases. Short-term exposure to wood smoke (WS) is partially known to alter the expression of human matrix metalloproteinases (MMPs), inflammatory cytokines, and tissue inhibitors of metalloproteinases (TIMPs). Accordingly, we investigated the effect of exposing guinea pigs to WS for two and four three-hour periods on different days. The daily content of particles reported by indoor pollution was produced by 60 g of pinewood. We analyzed the cell profile and collagen content in bronchoalveolar lavages (BAL). The mRNA expression of pro-inflammatory cytokines, MMPs, and TIMPs was studied in lung tissue. Cytokines and gelatinolytic activity were analyzed in BAL and serum. The results showed that total cells, macrophages, neutrophils, and collagen increased in BAL, whereas neutrophils and lymphocytes decreased. TGF-β1, TNF-α, IFN-γ, IL-1β, IL-6, IL-8, MMP-2, MMP-9, TIMP-1, and TIMP-2 were upregulated in lungs, downregulating IL-12. TNF-α, IFN-γ, TGF-β1, IL-1β, IL-6, and IL-8 were increased in BAL and serum, decreasing IL-12. Gelatinase activity was increased in serum. Thus, guinea pigs exposed to short-term domestic doses of WS overexpressed pro-inflammatory cytokines, MMPs, and TIMPs. These results are similar to ECM remodeling and pulmonary and systemic inflammation reported in humans.

## 1. Introduction

One ambient air polluting source is the smoke derived from biomass combustion, especially wood smoke (WS) derived from domestic incineration and forest fires [1]. WS is a global poor health risk factor that can cause oxidative stress, inflammation, and remodeling of the extracellular matrix (ECM). WS can elicit several lung diseases, such as bronchitis, pneumonia, acute respiratory failure, chronic obstructive pulmonary disease (COPD), and various systemic diseases [2,3,4,5,6]. Exposure to biomass smoke by indoor air pollution is strongly associated with COPD in the long term [2,3]. 

In forest fires, smoke is derived from the incineration of several plant species, which contain a complex mixture of toxic molecules, particles, and noxious gases, representing a significant risk of exposure to firefighters and ordinary people [5,6]. Consequently, it induces injuries and respiratory failure, accentuates the burn injury process, and induces cellular damage in the short term. The failure of affected organs is currently known as the leading cause of death in emergency burn care centers [2,3,4,5,6].

WS contains at least 400 compounds released during combustion, most of which are very similar to those present in cigarette smoke [7]. WS contains high levels of CO, CO_2_, nitrogen oxides, dioxins, particulate matter (PM_1–10_), and polycyclic aromatic hydrocarbons. The WS composition depends on the source of wood, the conditions of the incineration, and the combustion phase. This composition differs from the smoke released when biomass is incinerated, including coal, crops, leaves, or animal waste [6,8]. 

Although knowledge of the mechanisms that operate in WS toxicity is incipient, evidence derives from human volunteers exposed to controlled components, doses, and periods of WS. Pulmonary, cardiovascular, and systemic effects include inflammation and oxidative stress [8]. Similarly, there are several shreds of evidence in animal models, utilizing different animal species with very variable types of wood, times of exposure, and doses of WS [8,9]. However, very little is known about the role of matrix metalloproteinases (MMPs) and tissue inhibitors of metalloproteinases (TIMPs) [8,9,10].

The difficulty of exposing humans to whole components of WS and the variable conditions to which animals have been exposed support the use of a model that simulates the reported doses of WS due to indoor pollution for humans. It is beneficial to analyze the biochemical and pathophysiological alterations from the beginning and in short-term exposures. 

Consequently, we used a model developed in our laboratory [11,12,13] where guinea pigs are exposed to WS particles equivalent to those reported by indoor domestic exposure. The animals presented pulmonary inflammation and histological lesions, both in the airways and lung parenchyma. Oxidative stress [12] and alterations in respiratory mitochondrial complexes I and IV were ubiquitous [13]. Here, we studied how the short-term exposure to WS affected the expression and function of factors involved in ECM remodeling.

The animals were exposed to the smoke produced by 60 g of wood three hours per day, sacrificing them after 24 and 72 h. The mRNA expression of pro-inflammatory cytokines, MMPs, and TIMPs in the lung was evaluated. In addition to the cell profile and the total collagen content in BAL, we also studied the cytokines’ concentration and the gelatinase activity in the BAL and serum. 

## 2. Materials and Methods

### 2.1. Ethics Statement

The Ethics, Scientific, and Biosecurity Committees at Instituto Nacional de Enfermedades Respiratorias Ismael Cosío Villegas (INER), approved the present study with the protocol number B23-15, on 1 July 2015. 

### 2.2. Short-Term Model of Exposure to WS

Animals lived in a 12 h light/dark cycle in a room with 50–70% humidity and conditioned filtered air at 21 ± 1 °C. Ad libitum access to water and food was provided (Teklad Global 2018S; Harlan Laboratories; Madison, WI, USA). The experiments were conducted following the Guide for the Care and Use of Laboratory Animals. 

Two groups of 8 female guinea pigs (700–800 g) were exposed inside a chamber to the smoke produced by 60 g of pinewood in a period of 3 h per day, as previously described [11,12]. The amount of burnt wood was adjusted to set a continuous exposure of <80 ppm of CO to avoid hypoxic or ischemic changes.

The first experimental group was sacrificed after 24 h (two periods of exposure) and the second group 72 h after (four periods of exposure). Control animals (*n* = 8) were exposed to ambient air in a similar exposure chamber at the designated times and periods. 

Sodium pentobarbital (50 mg/kg body weight) was used to anesthetize animals with a single intraperitoneal injection. Serum was obtained by cardiac punction. Lungs were subject to BAL through a tracheal cannula using two flushes with 8 mL of sterile phosphate buffer saline (PBS) at 37 °C. We used a syringe pump system (Syringe Pump-Single Channel KL602; Beijing Kelly Med Co., Ltd., Beijing, China) to generate and maintain a constant pressure of 25 cm H_2_O. In some animals, the right lungs were used to confirm the histological analysis, whereas the left lungs were used to extract RNA [12,13].

### 2.3. Carboxyhemoglobin Analysis and WS Composition 

During WS exposure, the concentrations of O_2_, CO, CO_2_, PM_2.5,_ and PM_10_ particles were evaluated in the inhalation chamber, as previously described [12,13]. The monitored CO concentration in the inhalation chamber was maintained at <80 ppm during all exposures with a CO detector (MiniCO Responder Kit Dosimeter, Mine Safety Appliances Co., Pittsburgh, PA, USA). The WS exposure was evaluated using co-oximetry by the percent of blood carboxyhemoglobin (COHb%) [12,13]. 

### 2.4. Histology

For histology, a tracheal cannula and a syringe pump were used to fix the right lungs in situ, maintaining a pressure of 25 cm H_2_O, with phosphate-buffered 4% formaldehyde (pH 7.4) [12]. Lung tissues were processed for light microscopy and immunohistochemistry using conventional approaches after embedding in paraffin. Then, 6 mm tissue sections were stained with hematoxylin–eosin. Slides were analyzed using a Zeiss Axio Imager microscope. A Zeiss AxioCam MRc5 camera was used to capture and digitalize images. The Tab4Lab Document Software (Carl Zeiss AG, Oberkochen, Germany) was used for image analyses. Ten fields were horizontally screened (magnifications used were 4, 10, 20, and 40×) in hematoxylin–eosin-stained slides.

### 2.5. Cell Profile Analysis in BAL Fluid

To collect the cellular pellet, BAL were centrifuged at 4 °C for 10 min at 300× *g*. This pellet was resuspended in PBS, and viability was evaluated using trypan blue exclusion (0.4% trypan blue in PBS). Using an automated cell counter (Countess, Invitrogen, Carlsbad, CA, USA), the cell viability was determined and registered as the percentage of viable cells (the number of viable cells divided by total cells). Then, 2% carbowax (50% polyethylene glycol) and 50% ethyl alcohol were used to fix 100 µL of BAL cells. BAL cell numbers in leukocytes and the percentages of macrophage, neutrophil, lymphocyte, and eosinophil were quantified using the hematoxylin–eosin stain [12]. The BAL fluid was stored at −80 °C until their use. 

Serum was obtained from whole blood collected in sterile vacutainer blood collection tubes. Serum samples were separated after centrifugation at 5000× *g* 10 min at 4 °C and kept at −20 °C until examination. 

### 2.6. Total Collagen Content Measurement in BAL

According to the manufacturer’s instructions, all types of collagens in BAL were measured with the quantitative Sircol Soluble Collagen Assay (Biocolor Ltd., Carrickfergus, Northern Ireland, UK) [14]. The concentration of proteins in the cell culture medium was evaluated using the Bradford Assay Reagent (Bio-Rad Laboratories Inc., Hercules, CA, USA) [15]. The results were expressed as the mean ± SD of µg of collagen/mg of protein.

### 2.7. Gene Expression by qRT-PCR in Lung Tissue

Using SYBR Green, the gene expression of cytokines, MMPs, and TIMPs in lung tissue was performed by real-time quantitative reverse transcription-polymerase chain reaction (qRT-PCR) assays. All PCRs were normalized to the mRNA expression of GAPDH (Glyceraldehyde 3-phosphate dehydrogenase), utilizing previous standardization [16]. Treatments did not modify the ΔCt of the GAPDH mRNA significantly. A low variability was observed with the following Cts: 25.36 ± 2.78 in control, 22.69 ± 2.01 at 24 h, and 23.98 ± 1.68 at 48 h. Probes utilized for qRT-PCR are shown in Table 1. 

According to manufacturer’s instructions, 100 mg lung samples were used to extract total RNA using the Trizol Reagent (Invitrogen, Carlsbad, CA, USA) [17]. All qPCRs were performed in a mixture of 10 µL with 2 µg of cDNA and 8 µL of 2X PCR Master Mix (Applied Biosystems) [17]. The PCR conditions included 2 min at 94 °C for initial denaturation, followed by 40 cycles of 15 s at 94 °C and 60 s at 60 °C. Triplicates of three independent experiments were performed, and the results were presented as the mean ± standard deviation (SD) of the 2^−ΔCt^ of the target gene relative to GAPDH (ΔCT = Ct (a target gene) − Ct (a reference gene)). 

### 2.8. Quantification of Cytokines in BAL and Serum

Cytokines in the BAL and serum were quantified with enzyme-linked immunosorbent assays (ELISA) following the manufacturer’s instructions of the following kits for the guinea pigs: TNF-α (MBS9303082; MyBioSource, San Diego, CA, USA), IFN-γ (MBS701377; MyBioSource, San Diego, CA, USA), TGF-β1 (CSB-E06773GU; CUSABIO TECHNOLOGY LLC; Houston, TX, USA), IL-1β (MBS765173; MyBioSource, San Diego, CA, USA), 1L-6 (MBS269054; MyBioSource, San Diego, CA, USA) IL-8 (MBS282965; MyBioSource, San Diego, CA, USA), IL-12 (MBS704591; MyBioSource, San Diego, CA, USA).

### 2.9. Gelatinolytic Zymography Assay

The gelatin zymography quantifies the relative amounts of active and inactive gelatinases (zymogen) in soluble-aqueous samples by measuring the hydrolysis of gelatin (substrate in the gel). The enzymes gelatinase A (MMP-2) and gelatinase B (MMP-9) were fractionated on SDS-PAGE. After Coomassie staining, gelatin hydrolysis generates white bands for zymogen or active forms of the enzymes [18]. Briefly, cells were kept in the Ham’s F-12 Nutrient Mixture (F-12) without FBS for 24 and 48 h, the conditioned media were collected, and the Bradford method was used to measure protein [15]. Samples containing 10 µg of protein were mixed with an equal volume of sample buffer and resolved in non-reducing 7.5% SDS-PAGE with gelatin 1 mg/mL as a substrate (Cat. No. G-8150; Lot. 63H06591; Sigma, St. Louis, MO, USA). Conditioned media from human lung fibroblasts were used as a positive control for MMP-2. The MMP-9 positive control was obtained from U2-OS human cells.

### 2.10. Statistical Analysis

Results are presented as the mean ± SD, obtained from three independent experiments made in triplicate. Differences between the two groups were assessed using an unpaired Student’s t-test. In comparison, analyses among more than two groups were performed using one-way analysis of variance (ANOVA), followed by Tukey–Kramer post hoc test at 95% significance. All analyses were performed using GraphPad Prism v. 6.1 software (GraphPad Software, Inc., San Diego, CA, USA). A *p* < 0.05 was considered statistically significant.

## 3. Results

### 3.1. WS Composition and Carboxyhemoglobin (COHb) Analysis

Table 2 shows the concentrations of CO_2_, O_2_, and PM_10_, and PM_2.5_ particles measured in the exposure chamber. These were similar to WS concentrations found at rural areas homes of people exposed throughout Mexico [11] and similar to our previous short-exposure and chronic models of exposure to WS in guinea pigs [12,13].

The percentage of plasma COHb measured the exposure to WS in the animals and was increased after 24 and 72 h (Table 2). There was no mortality observed in this model. Additionally, bodyweight was not modified, and food intake did not change between study groups. 

### 3.2. Histological Analysis

**Figure 1:**
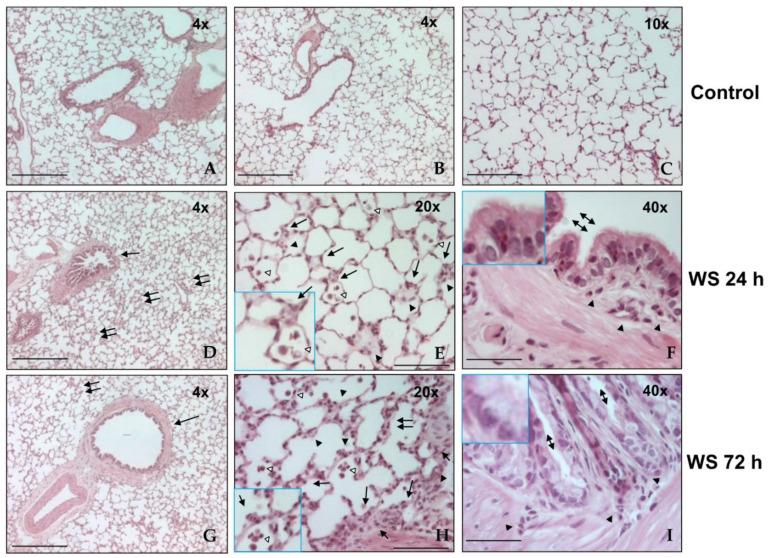
Representative photomicrographs of histological lung sections from WS-exposed guinea pigs and controls (*n* = 8). Controls (**A**–**C**), after 24 h (**D**–**F**), and after 72 h (**G**–**I**) of exposure to WS. The bottom-left inserts in panels **E** and **I** show intralveolar and alveolar macrophages (empty arrow) and polymorphonuclear leucocytes (arrow). Upper-left inset in panels in **F** and **I** display goblet cell hyperplasia. Hematoxylin–eosin stain. Original magnification, panels **A**, **B**, **D**, and **G**, 4×; panel **C**, 10×; panels **E** and **H**, 2×; panels **F** and **I**, 40×. Scale bars = 100 µm.

Figure 1 shows photomicrographs of lung histologies of WS-exposed and control guinea pigs. Controls are shown in Figure 1A–C; all images show the typical appearance of large and small airways and lung parenchyma. Guinea pigs exposed to WS exhibited mild inflammation and damage in airways and pulmonary parenchyma after 24 and 72 h (Figure 1D–G). The most relevant histological changes consisted of mild thickness in bronchial and bronchiolar walls (arrow, Figure 1D,G), with localized areas of inflammation in the lung parenchyma (double arrow, Figure 1D,G). These changes included inflammatory infiltration of foamy macrophages in alveolar spaces (empty arrowhead, Figure 1E,H) and septum (arrow, Figure 1E,H), and increased polymorphonuclear leukocytes on alveolar walls (arrowhead, Figure 1F,H), the bronchial and bronchiolar epithelium, and walls (arrowhead, Figure 1F,I). Bronchial and bronchiolar epithelium with notorious goblet cell hyperplasia was also noted (double arrowhead, Figure 1F,I). This histological analysis corroborated the observations documented previously in this model [12].

### 3.3. WS Induces an Increase in Macrophages and Neutrophils and Diminished Lymphocytes and Eosinophils in BAL

**Figure 2:**
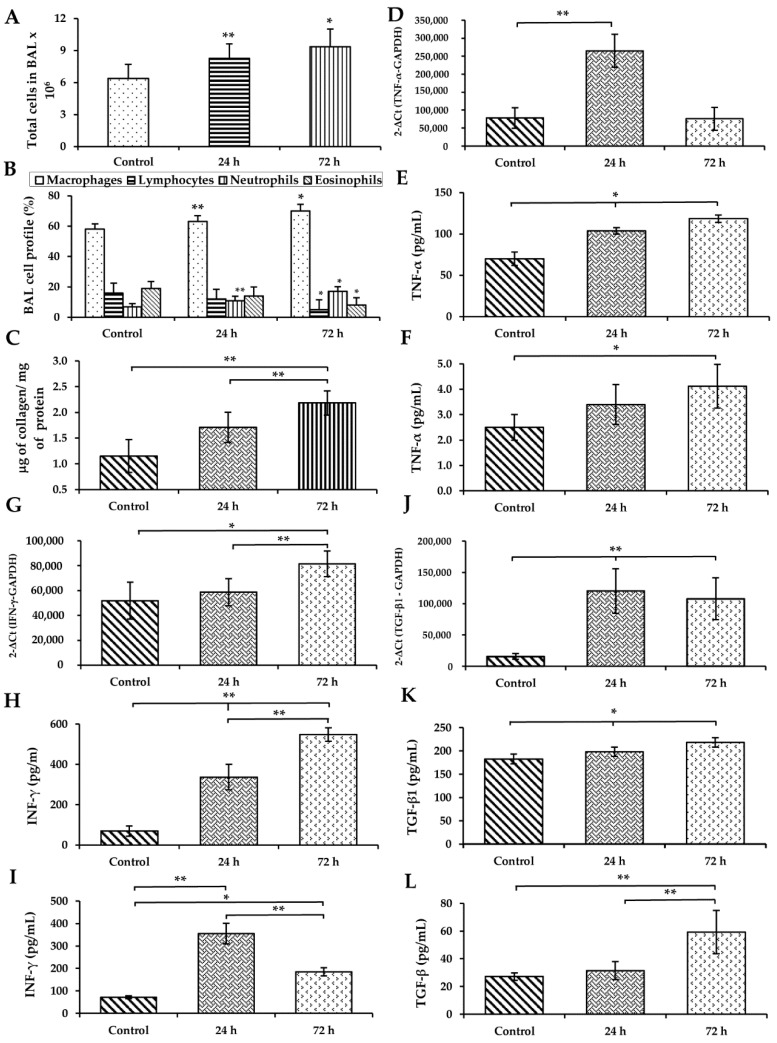
Effect of short-term exposure to WS on total cell number (**A**), differential cell count (**B**), and total soluble collagen (**C**) in BAL; mRNA expression levels of TNF-α (**D**), IFN-γ (**G**), and TGF-β1 (**J**) in the lung; protein concentration of TNF-α in BAL (**E**) and serum (**F**), IFN- γ in BAL (**H**) and serum (**I**), and TGF-β1 in BAL (**K**) and serum (**L**). Total cells and the number of macrophages, neutrophils, eosinophils, and lymphocytes were analyzed. Total collagen synthesis was measured with Sircol reagent in BAL fluid recovered from guinea pigs exposed to WS and filtered-ambient-air-exposed controls. qRT-PCR was used to detect the mRNA levels of expression, and ELISA was employed to detect the protein levels in BAL and serum (*n* = 8). Statistical analyses were one-way analysis of variance (ANOVA), followed by Tukey–Kramer post hoc test. * *p* < 0.05; ** *p* < 0.01 compared with the control.

A differential cell count was carried out to assess the changes in total and inflammatory cells in BAL induced by WS exposure. The total number of leucocytes recovered in BAL was higher in WS-exposed animals after 24–72 h compared with control animals (*p* < 0.05 and *p* < 0.01, respectively; Figure 2A). The differential cell count is shown in Figure 2B; macrophages increased at all study times and were the prevalent population observed in WS-exposed animals. Similarly, neutrophils were increased at 24–72 h (*p* < 0.05 and *p* < 0.01, respectively; Figure 2B), while lymphocytes and eosinophils decreased at 72 h (*p* < 0.05 and *p* < 0.01, respectively; Figure 2B). The cell viability of recovered cells was 95.5 ± 3.2% in animals at different study times and was evaluated with trypan blue.

### 3.4. WS Induces an Increase in the Total Collagen Content in BAL 

Collagen content in BAL is a measurement of the new total collagen secreted to the extracellular medium. This quantification includes all types of collagens. Relative to controls, collagen was increased in BAL of guinea pigs exposed to short-term WS after 24 and 72 h (*p* < 0.01; Figure 2C).

### 3.5. WS Induces the Upregulation of Cytokines in the Lung and Increases Their Levels in BAL and Serum

Relative to controls, the qRT-PCR analysis showed a significant increase in the expression of TNF-α mRNA in the lungs of WS treated guinea pigs at 24 h (*p* < 0.01; Figure 2D). Furthermore, its protein was increased after 24 and 72 h in BAL (*p* < 0.05; Figure 2E) and in serum after 72 h (*p* < 0.01; Figure 2F). 

The lung mRNA and BAL protein expression of IFN-γ were upregulated by WS (*p* < 0.05; Figure 2G) at 72 h (*p* < 0.01; Figure 2H) in comparison to controls. In serum, the elevated IFN-γ expression was significant after 24 and 72 h (*p* < 0.01; Figure 2I).

The gene expression of TGF-β1 was upregulated in WS samples relative to controls at 24 and 72 h (Figure 2J). The protein increased in BAL after 24 and 72 h (*p* < 0.01; Figure 2K) and at 72 h in serum (*p* < 0.01; Figure 2L). 

**Figure 3:**
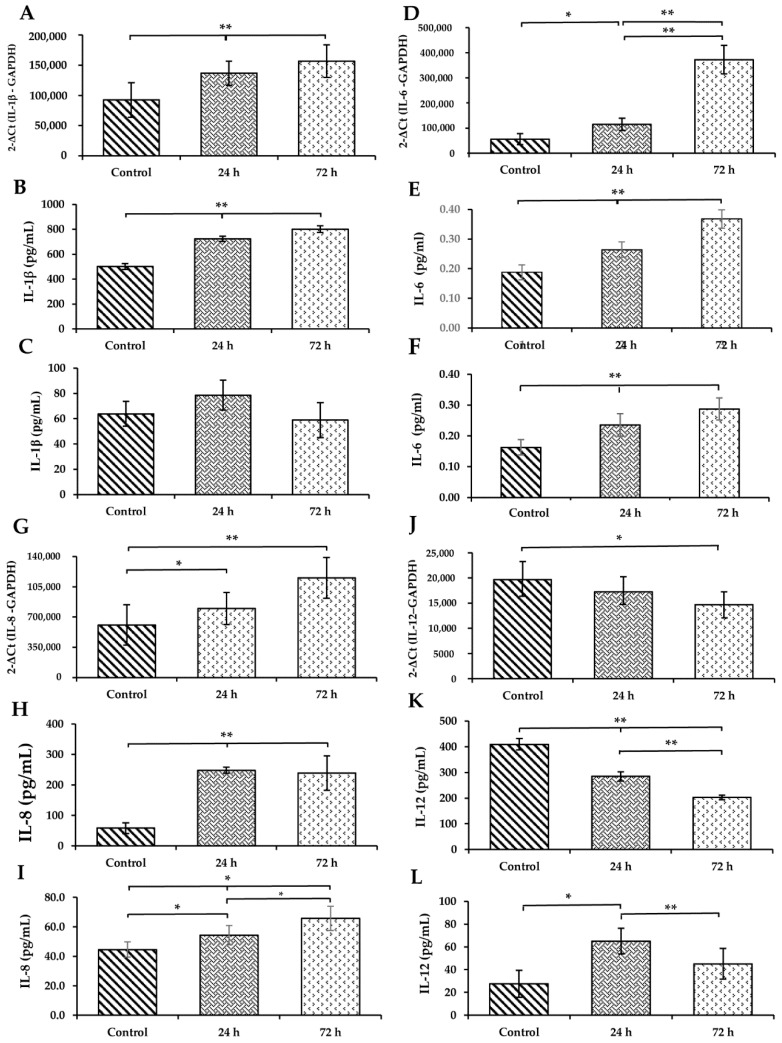
Effect of short-term WS in mRNA expression levels of IL-1β (**A**), IL-6 (**D**), IL-8 (**G**), IL-12 (**J**) in the lung, and protein concentration of IL-1β in BAL (**B**) and serum (**C**), IL-6 in BAL (**E**) and serum (**F**), IL-8 in BAL (**H**) and serum (**I**), and IL-12 in BAL (**K**) and serum (**L**). qRT-PCR was used to detect the mRNA levels of expression, and ELISA was employed to detect the protein levels in BAL and serum (*n* = 8). Statistical analyses were one-way analysis of variance (ANOVA), followed by Tukey–Kramer post hoc test. * *p* < 0.05; ** *p* < 0.01 compared with the control.

Compared to controls, WS upregulated the expression of IL-1β after 24 and 72 h (*p* < 0.01; Figure 3A). Their protein was also increased in BAL at identical times (*p* < 0.01; Figure 3B). No significant changes were found for serum IL-1β (Figure 3C). 

WS induced the upregulation of the expression levels of IL-6 in lung mRNA (*p* < 0.01; Figure 3D), and their protein in BAL (*p* < 0.01; Figure 3E) and in serum (*p* < 0.01; Figure 3F) after 24 and 72 h. 

In a similar way to IL-6, WS induces a significant increase in the IL-8 mRNA expression after 24 and 72 h (*p* < 0.05 and *p* < 0.01, respectively; Figure 3G). The IL-8 protein levels in BAL (*p* < 0.01; Figure 3H) and in serum (*p* < 0.01; Figure 3I) were also elevated at 24 and 72 h. 

The mRNA expression level of IL-12, opposite to the other cytokines evaluated, was significantly downregulated at 72 h (*p* < 0.05; Figure 3J). Similarly, its protein decreased in BAL after 24 and 72 h (*p* < 0.01; Figure 3K). However, the serum level of IL-12 was increased after 24 and 72 h (*p* < 0.05 and *p* < 0.01, respectively; Figure 3L) in guinea pigs exposed to WS compared to those exposed to ambient filter air.

### 3.6. WS Upregulates MMP-2 and MMP-9 and Increases the Activity of MMP-2 

**Figure 4:**
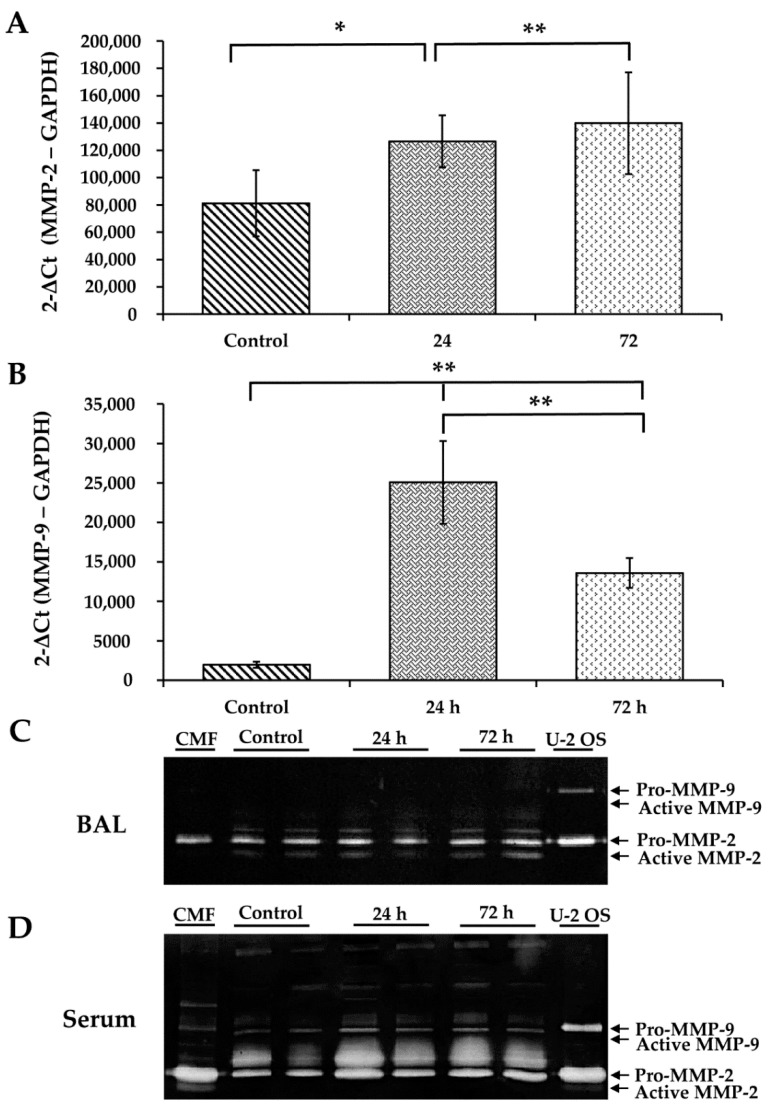
The effect of WS on MMP-2 and MMP-9 mRNA expression in lung and gelatinolytic activity in BAL and serum. Expression of MMP-2 (**A**) and MMP-9 (**B**) mRNAs in lung and gelatinolytic activity in BAL (**C**) and serum (**D**). qRT-PCR detected the mRNA levels of MMP-2 and MMP-9. Gelatinolytic zymography detected the gelatin activity of MMP-2 and MMP-9 in BAL and serum of WS treated guinea pigs (*n* = 8). * *p* < 0.05, ** *p* < 0.01 compared with the control.

qRT-PCR analysis showed a significant increase in MMP-2 (*p* < 0.05 and *p* < 0.01, respectively; Figure 4A) and MMP-9 (*p* < 0.01; Figure 4B) mRNA expression relative to controls, after 24–72 h. 

The serum gelatinolytic activities of MMP-2 and MMP-9 were assayed by zymography. MMP-2 (Figure 4D), but not MMP-9, was increased by exposure to WS (Figure 4D). The clear bands of the zymogen Pro-MMP-2 (72 kDa) and its active enzyme MMP-2 (62 kDa) were increased in comparison with the control after 24–72 h (Figure 4D). However, in the case of MMP-9, the clear bands corresponding to the zymogen Pro-MMP-9 (92 kDa) and its active enzyme MMP-9 (82 kDa) did not change (Figure 4C). The culture medium of the U2-OS cell line was used as a positive control and showed the clear bands corresponding to the tested MMPs (Figure 4D). 

### 3.7. WS Upregulates the mRNA Expression Levels of TIMP-1 and TIMP-2

**Figure 5:**
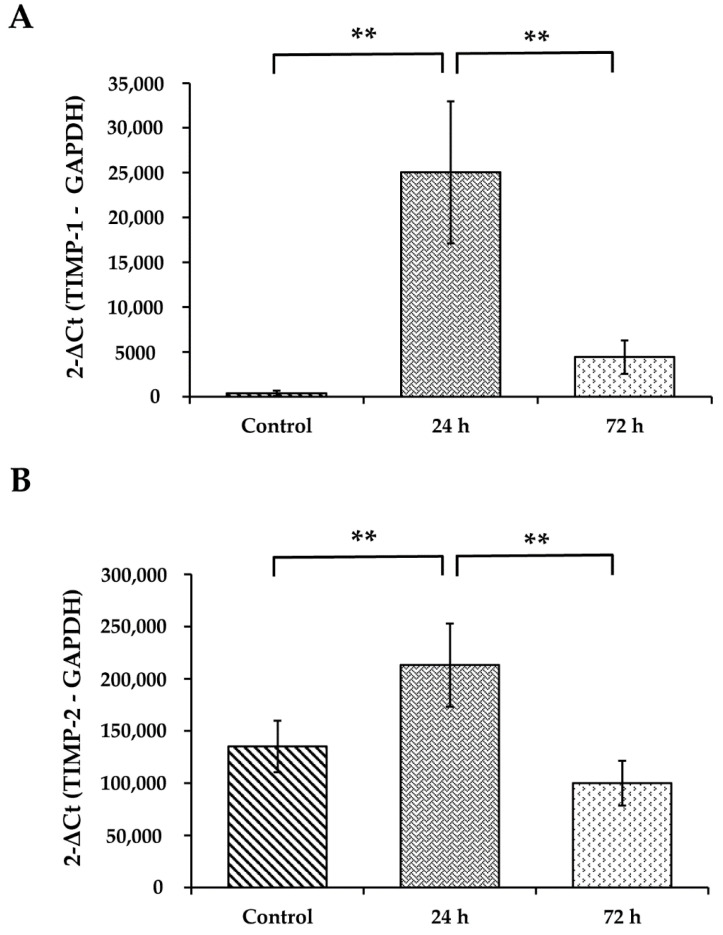
Effect of WS in TIMP-1 (**A**) and TIMP-2 (**B**) mRNA expression in lung tissue. qRT-PCR was used to detect the mRNA levels of TIMP-1 and TIMP-2 (*n* = 8). ** *p* < 0.01 compared with the control.

TIMP-1 and TIMP-2 are the specific tissue inhibitors of MMPs. WS induced the upregulation of the mRNA of both TIMP-1 (*p* < 0.01; Figure 5A) and TIMP-2 (*p* < 0.01; Figure 5B) after 24 h of incubation when compared with the control. 

## 4. Discussion

This study focused on analyzing pro-inflammatory mediators and the molecules involved in the ECM remodeling induced by WS. The features observed here are consistent with the upregulation of the mRNAs of several inflammatory cytokines, MMPs, and TIMPs in the lungs. The cytokine levels in BAL and serum were also increased, accompanied by the increment in the total collagen content in BAL and the gelatinase activity in serum at 24 and 72 h. The augmented cytokines are involved in the pulmonary and systemic inflammatory response and some in the ECM remodeling [4,9,10,19,20,21,22,23,24]. 

The findings correlate with reports of WS-induced damage in guinea pigs [12,13] and with the systemic and local lung effects [20,21,22]. The BAL macrophages and neutrophils increase, and the decreases in lymphocytes and eosinophils were confirmed, as alterations in the histopathology morphological changes in airways and lung parenchyma, and the hyperplasia of goblet cells [12].

The increase in total cells, macrophages, and neutrophils in BAL after 24 and 72 h has been similarly observed in other experimental models [24]. However, the quantity and kind of WS were unknown, and the administration of WS was different from our model. Firefighters and people exposed to WS during fires [5] and healthy human volunteers with controlled exposure to WS and other particles have increased neutrophils in BAL and serum 24 h after exposure [25,26,27,28]. 

The increase in macrophages and neutrophils is probably involved in foreign particles clean-up. This is pobably to avoid the translocation of toxic particles to the pulmonary interstitium and the systemic vasculature [5,26,29,30,31,32].

Macrophages also participate in the inflammatory response to WS. These cells present antigens and secret chemoattractants, pro-inflammatory mediators, proteinases, MMPs, and TIMPs. After WS exposure, the secreted factors probably remodel the ECM, and regenerate and repair tissue [5,26,27]. Additionally, the macrophages secrete chemoattractants to neutrophiles and inductors of the systemic inflammatory response [27,28,30,32,33]. 

Two macrophage types have been recognized, called M1 and M2. The M1 macrophages are regulated by the Th-1 type immune cells, mainly through IFN-γ and IL-12, which increase in the BAL. At the same time, M2 induces Th2-type activation by cytokines such as IL-4 and IL-13 or immunoregulators such as IL-10 [22,28]. INF-γ is one of the primary activators of M1 macrophages. These cells release inflammatory cytokines and chemokines such as TNF-α, IL-6, and IL-12, which could initiate the inflammatory response in response to WS in our model. In bronchial cells and mice exposed to WS, IL-8, and MMP-1, MMP-9 and MMP-12 were overexpressed, while TIMP-1 was downregulated, activating the p38 and JNK signaling pathways [5,27]. This is a similar response to that observed in our model. 

Similarly, other studies on the acute effect of WS particles in firefighters exposed to natural disasters showed similar effects in the sputum of these subjects. Specifically, macrophages loaded with carbonaceous particles increased, as well as the presence of pro-inflammatory mediators such as IL-6 and IL-8, among others [31]. 

Additional studies carried out in firefighters have shown, in addition to DNA breaks, an increase in serum TNF-α, IL-6, IL-8, and C-reactive protein, with a decrease in the serum levels of ICAM-1 and sVCAM-1 [5,32]. Moreover, IL-1β was increased in healthy volunteers exposed to WS particles [26]. These studies show the production of pro-inflammatory mediators similar to those we have described, although they also show other effects such as genomic mutations. On the other hand, the significant increases in TNF-α, IL-6, and IL-8 may lead to potent induction of systemic inflammation, potentially damaging other organs [5,33]. 

WS increases serum concentrations of TNF-α, IFN-γ, TGF-β1, IL-1β, IL-6, and IL-8 at 24 and 72 h. These factors also participate in the modulation of the immune response by the effect of several inhaled particles, indicating that these cytokines act quickly in the inflammatory process [34]. IFN-γ is one of the first cytokines to be activated within the immune system. The damage produced to the alveolar epithelium, especially to type II pneumocytes, induces an increase in IL-1β, which in combination with TNF-α acts as a potent anti-inflammatory cytokine. All these cytokines are secreted by macrophages in response to WS [33], while TNF-α and TGF-β1 are secreted by type II pneumocytes [34]. 

These cytokines have diverse several modulatory effects in other pulmonary cells, including fibroblasts, neutrophils, bronchial and bronchiolar epithelial cells, and goblet cells, which contributed to the development of lesions caused by WS [35,36]. Instead, a central source of IFN-γ in the lung are the lymphocytes, these lymphocytes could be an important source of this cytokine, also contributing to the inflammatory process [37,38].

TGF-β1 expression increased gradually at 24 and 72 h. When this cytokine is activated, it could have a regenerative and reparative effect on the ECM, inducing MMPs and TIMPs. TGF-β1 coordinately regulates the remodeling of all the components of the ECM and the expression of MMPs and TIMPs. In a possible attempt to regenerate and repair lung tissue, its effect would be contrary to TNF-α, but analogous to IFN-γ, which is the most protective at the inflammatory level [29,30,37,38]. The increase in TGF-β1 in the serum reflects a systemic effect in the remodeling of ECM in the various tissues, a fact known to occur as an effect of WS in humans [32,33,39]. The most important sources of TGF-β1 are the macrophages, type II pneumocytes, and bronchial and bronchiolar epithelial cells [29,30,39]. 

Our model’s expression of MMP-2, MMP-9, TIMP-1, and TIMP-2 increased, reflecting the ECM remodeling of both basement membranes and the interstitium. This remodeling effect also shows a systemic component since, in the gelatinolytic zymography, the MMP-2 and MMP-9 serum activities were elevated by WS. This effect can be considered a reflection of the ECM remodeling. Even when in BAL, no increase in these enzymes was noted [29,30]. 

At the physiological level, the effect of WS on lung ECM remodeling can be seen in the increase in total collagen synthesized in BAL. This increase is also related to tissue damage produced by WS, as reported in other studies [19,20,21,40].

Particles PM_10_ are released by incineration of a variety of biomasses. Recent evidence showed that indoor stove incineration of charcoal and wood released PM_10_ containing high metal (Zinc) and polycyclic aromatic hydrocarbons. These compounds elicit DNA damage, oxidant stress, alterations in the cell cycle, and other cytotoxic and genotoxic effects in human type II cells [41]. These findings support the fact that PM_10_ are important indoor noxious pollutants and part of the inflammation and the ECM remodeling alterations observed in our model. However, compared with in vitro approaches testing isolated particles, the reported in vivo effects are probably due to the biological response to different components of biomass smoke in synergy. 

Finally, considering the evidence contained in this work and those previously published [12,13], various similarities exist concerning the presence of lung inflammation, local and systemic pro-inflammatory mediators, oxidant stress, and ECM remodeling. Thus, our model could help evaluate strategies to minimize the effects caused by acute exposure to WS since we use doses reported in poorly ventilated rooms in rural Mexico [11].

## 5. Conclusions

With similar doses to indoor pollution measurements, acute exposure to WS induces the overexpression of some pro-inflammatory cytokines, gelatinases (MMP-2, and MMP-9), and TIMPs (TIMP-1 and TIMP-2) in BAL and serum. Total collagen secreted in BAL also increases after 24 and 72 h in guinea pigs. This cellular response relates to the development of lung and systemic inflammatory processes, favoring acute lung damage and tissue remodeling. Furthermore, the increase in the concentration of cytokines in serum could be associated with the development of systemic inflammation, and consequently, the damage caused by WS may affect other organs. Finally, the increases in macrophages and neutrophils observed in BAL would be involved, in part, in the tissue repair mechanisms associated with the ECM turnover due to the cytokines, MMPs, and TIMPs that these cells secrete.

## Figures and Tables

**Table 1:** List of primers for RT-qPCR.

Gene	Primer Sequence (5′–3′)	Product Size (bp)
TNF-α	Forward AACTCCAGCCGGTGCCTAT Reverse GTTCAGCAGGCAGAAGAGGATT	81
INF-γ	Forward GGCCATCCAGAGGAGCATAG Reverse CCATGCTGCTGTTGAAGAAGTTAG	68
TGF-β1	Forward GCGGCAGCTGTACATCGA Reverse GGCTCGTGAATCCACTTCCA	57
IL-1β	Forward CTTGAGGACTGGACCTTTTGC Reverse TCGTCACTGTGGTAAGCTGT	80
IL-6	Forward GTTCAGCACGACTTCACAGC Reverse TGTGAAGCAGAGGTTGTTGGT	212
IL-8	Forward TAGGGTGGCAGATTTAACTCA Reverse TCAGGAATTGGCTTGCTAC	112
IL-12	Forward AAAACCAGCACCGTGAAAGC Reverse AAAACCAGCACCGTGAAAGC	296
MMP-2	Forward CTACCCTTGTACCACCATCGA Reverse TTAGCTGACCGTCACCAATC	462
MMP-9	Forward GTGACACCGCTCACCTTCAC Reverse GCGTGTGCCAGTAGACCATC	122
TIMP-1	Forward GATCGGATGCCTTGGGACAT Reverse TTCTGGGACGGGTGGAAGTA	326
TIMP-2	Forward GACCTGTCTGACGCCCTACCTCTT Reverse ATGGGAACCCCATCAAGCGA	358
GAPDH	Forward TCAGAGGGCTCCCTCAAAG Reverse CGCTGTTGAAGTCACAGGAC	70

**Table 2:** WS composition and carboxyhemoglobin (COHb) analysis.

Molecule	Concentration in Chamber
CO_2_	0.31 ± 0.13%
O_2_	19.8 ± 0.15%
PM_10_	479 ± 45 mg/m^3^
PM_2.5_	361 ± 32 mg/m^3^
Plasma COHb	(%)
Control	3.36 ± 2.12
WS 24 h	12.96 ± 4.91 **
WS 72 h	14.36 ± 5.89 **

Data were analyzed in guinea pigs exposed to WS and controls exposed to filtered ambient air (*n* = 8). Statistical analyses were one-way analysis of variance (ANOVA), followed by Tukey–Kramer post hoc test. ** *p* < 0.01 compared with the control.

## Data Availability

Data related to this study can be requested from Martha Montaño (corresponding author) or Carlos Ramos (first author).
